# The Search for Dietary Supplements to Elevate or Activate Circulating Paraoxonases

**DOI:** 10.3390/ijms18020416

**Published:** 2017-02-15

**Authors:** José M. Lou-Bonafonte, Clara Gabás-Rivera, María A. Navarro, Jesús Osada

**Affiliations:** 1 Departamento de Farmacología y Fisiología, Facultad de Ciencias de la Salud y del Deporte, Huesca E-22002, Spain; mlou@unizar.es; 2 Instituto de Investigación Sanitaria de Aragón, Universidad de Zaragoza, Zaragoza E-50009, Spain; angelesn@unizar.es; 3 Instituto Agroalimentario de Aragón, Centro de Investigación y Tecnología Agroalimentaria, Universidad de Zaragoza, Zaragoza E-50013, Spain; 4 Centro de Investigación Biomédica en Red de Fisiopatología de la Obesidad y Nutrición, Instituto de Salud Carlos III, Madrid E-28029, Spain; clarisgr_@hotmail.com; 5 Departamento de Bioquímica y Biología Molecular y Celular, Facultad de Veterinaria, Zaragoza E-50013, Spain

**Keywords:** paraoxonase 1, paraoxonase 2, catechins, genistein, plant extracts, atherosclerosis

## Abstract

Low levels of paraoxonase 1 (PON1) have been associated with the development of several pathological conditions, whereas high levels have been shown to be anti-atherosclerotic in mouse models. These findings suggest that PON1 could be a good surrogate biomarker. The other members of the family, namely PON2 and PON3, the role of which has been much less studied, deserve more attention. This paper provides a systematic review of current evidence concerning dietary supplements in that regard. Preliminary studies indicate that the response to dietary supplements may have a nutrigenetic aspect that will need to be considered in large population studies or in clinical trials. A wide range of plant preparations have been found to have a positive action, with pomegranate and some of its components being the best characterized and *Aronia melanocarpa* one of the most active. Flavonoids are found in the composition of all active extracts, with catechins and genistein being the most promising agents for increasing PON1 activity. However, some caveats regarding the dose, length of treatment, bioavailability, and stability of these compounds in formulations still need to be addressed. Once these issues have been resolved, these compounds could be included as nutraceuticals and functional foods capable of increasing PON1 activity, thereby helping with the long-term prevention of atherosclerosis and other chronic ailments.

## 1. Introduction

Atherosclerosis is a chronic disorder of the arterial wall that starts with the formation of fatty streaks and gradually evolves into atherosclerotic plaques [1]. Blood high-density lipoprotein (HDL) levels are inversely correlated with atherosclerosis [2], with this beneficial effect of HDL being partly attributed to its antioxidant properties mediated by paraoxonase 1 (PON1) [3,4] or platelet-activating factor acetylhydrolase (PAF-AH) [5].

**Figure 1:**
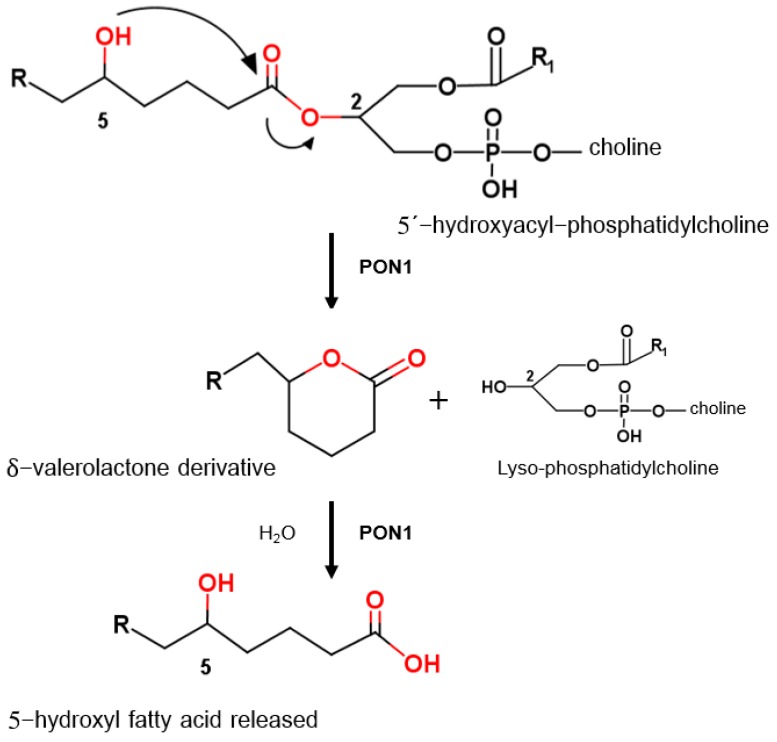
Proposed biological role of paraoxonase 1. Adapted from [7], reproduced with permission from the American Society for Biochemistry and Molecular Biology. Although 5-hydroxy fatty acids are not frequent in normal physiology, they appear during inflammatory processes in macrophages and dendritic cells due to the action of cytochromes P-450 and 5′-lipooxygenase [9]. The extent to which they can esterify to form phosphatidylcholine is unknown.

The *PON1* gene, together with the genes for paraoxonases 2 and 3, form a gene family located on the long arm of human chromosome 7 (7q21-22). PON1 is synthesized in the liver and secreted into the serum as an HDL-associated protein [6]. As an antioxidant enzyme carried by HDL, it can hydrolyze lipid peroxide in lipoproteins as a result of its lipolactonase activity (Figure 1) [7,8], thereby decreasing oxidative stress in serum lipoproteins, macrophages, and atherosclerotic lesions.

**Figure 2:**
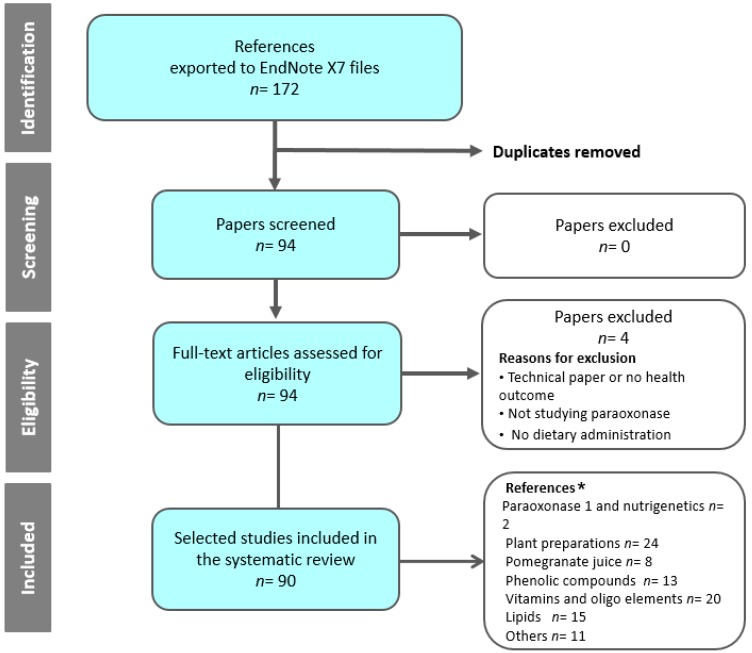
Flow chart displaying the steps followed to select the references considered. EndNote X7 (Thomson Reuters: New York, NY, USA, 2016). * Some references may appear in more than one section of the review.

In light of the above, PON1 could be partly responsible for the anti-inflammatory and antiatherogenic properties of HDL molecules in blood [10]. Serum PON1 activity has been inversely associated with the risk of cardiovascular disease [11] and has been found to be decreased in several situations associated with atherosclerosis and oxidative stress [12]. Direct proof of its effects on atherosclerosis development was obtained from *Pon1*-deficient mice [13] and their counterparts overexpressing human *PON1* gene [14]. As such, PON1 plays a major role in the protective role of HDL, and its status is considered to be one of the determinants that predispose people to coronary artery and other diseases [12]. Since PON1 undergoes inactivation by oxidative stress, its activity needs to be preserved [10]. Given the panoply of positive actions of PON1, pharmacological and nutritional modulation of PON1 activity and/or gene expression could constitute a useful approach to the prevention of cardiovascular and other diseases, such as diabetes, Alzheimer’s disease, chronic renal failure, and chronic liver impairment [15]. A recent review concerning PON1 and the Mediterranean diet analyzed the impact of this diet [16]. Dietary supplements also represent a new approach to enhancing population-based health [17]. In this work, we have searched for evidence of the effect of dietary supplements and plant extracts on PON1 by way of a strategy following systematic review guidelines [18]. As displayed in Figure 2, a search in PubMed using the keywords (paraoxonase and dietary supplements and paraoxonase and plant extracts), or (“aryldialkylphosphatase” [Mesh]) AND (“Plant Extracts” [Mesh] OR “Dietary Supplements” [Mesh]) identified 172 hits between November 1945 and 04 October 2016. The search was refined by eliminating duplicate documents. The 94 papers obtained were critically reviewed to verify whether they analyzed PON1 and dietary administration. Documents that failed to meet this criterion were excluded. Thus, this review covers the studies related to the effects of dietary supplements and PON1 published in 90 papers.

## 2. PON1 and Nutrigenetics

The interindividual variation in the response of PON1 activity to dietary supplements is partly influenced by genetic factors, such as polymorphisms in the *APOE* or *PON1* genes, as indicated by two experimental studies. In the first study, administration of a diet containing 2 g/kg quercetin for six weeks resulted in lower hepatic mRNA and protein levels of PON1 in *APOE4* than in *APOE3* transgenic mice. These results indicate that PON1 is differentially regulated in response to the APOE genotype [19]. The second study had a cross-over design and was intended to determine the effect of 250 mL of antioxidant-rich orange plus 250 mL of blackcurrant juice and 15 mg vitamin E supplement for 28 days on PON1 activity, although no changes were observed in patients with peripheral arterial disease. However, a gene–diet interaction was observed between dietary treatment and the *PON1* genotype, with PON1 activity increasing after consumption of juices alone in patients carrying the PON1L55 allele [20].

## 3. Plant Preparations

A great deal of experimental work has been carried out on the effect of different plants on PON1, with a large variety of models also having been tested. In this regard, mice and rats are the most widely used models for testing the effect of plant extracts. For example, *Eucommia ulmoides* Oliver leaf extract equivalent to 1% dried whole leaf (1.87 g of extract/kg of diet) was administered for six weeks to type 2 diabetic C57BL/KsJ-db/db mice. This extract elevated plasma PON1 activity compared with the control group [21]. A similar effect was observed by Saha et al., who reported that PON1 activity in serum and liver increased after administration of an aqueous *Murraya koenigii* extract at doses of 75 and 150 mg/kg body weight in streptozotocin-induced diabetic mice [22]. Likewise, supplementation of grape seed extracts recovered the decreased PON1 activity found in streptozotocin-induced diabetic rats [23]. In contrast, El-Beshbishy et al. did not find recovery of PON1 activity upon administration of a daily dose of 500 mg/kg of ethanolic extracts from *Morus alba* L. (Egyptian mulberry) root bark in hypercholesterolemic rats [24].

Red wine polyphenol extract supplementation to the drinking water of heterozygous *Cbs*-deficient mice (a murine model of hyperhomocysteinemia) for four weeks increased hepatic *Pon1* expression and hepatic and plasma PON1 arylesterase activities [25]. Administration of a 1.25% (*w*/*w*) anthocyanin-rich extract from black elderberry (*Sambucus nigra*, 13% anthocyanins) containing cyanidin 3-sambubioside and cyanidin 3-glucoside (9.8% and 3.8%, *w*/*w*, respectively) significantly increased serum PON1 arylesterase activity in 10-week-old male *Apoe*-deficient mice after six weeks [26]. In this model, 0.05% dietary Chokeberry extract was also found to increase serum PON1 activity [27]. According to an analysis of doses used and the magnitude of the observed effect reflected in Table 1, the *Aronia melanocarpa* (chokeberry) extract was the most effective at increasing PON1 activity. It has been proposed that the phenolic compounds contained therein, especially anthocyanins, phenolic acids, proanthocyanidins, and flavonols, might be involved in this effect.

When Jaiswal et al. explored the effect of onion extract and flavonoids (quercetin and catechin) on the regulation of PON1 expression in male Wistar rats subjected to mercuric chloride-induced oxidative insult, they observed that onion extract significantly attenuated the adverse effects of HgCl_2_ by upregulating PON1 activity. Quercetin and, to a lesser extent, catechin induced similar effects [28]. In this animal model, the administration of *Aronia melanocarpa* juice statistically restored the PON activity of rats on a high-fructose and unsaturated fat diet [29]. Cornelian cherry, as a rich source of phenolic compounds, had a stimulating effect on PON1 activity in both the brain tissue and plasma of Wistar rats consuming control, high-fructose, and high-fat diets [30]. In rats with myocardial ischemia, treatment with flaxseed in combination with exercise recovered the decreased PON1 and HDL levels, whereas administration of flaxseed alone had no effect [31]. In a rat ischemia–reperfusion model, significant decreases were observed in the serum PON1 levels of animals given ginsenoside despite the decrease in malonyldialdehyde [32]. In contrast, the decrease of PON1 and arylesterase activities in arthritis-induced rats was restored after treatment with soy protein and isoflavone (genistein and daidzein) for 50 days [33]. Likewise, 2% (dry *w*/*w*) diet supplementation with pulp of *Euterpe oleracea* Mart also increased PON1 activity in female Fischer rats fed standard chow (AIN-93 M) or hypercholesterolemic (25% soy oil and 1% cholesterol) diets [34]. The inclusion of 28% avocado in the diet increased PON1 activity in Wistar male rats [35].

Supplementation of the ethyl acetate extract of *Vigna unguiculata* leaves (150 mg/kg body weight) normalized the decreased PON1 expression of rabbits fed a diet containing 1% cholesterol and 0.5% cholic acid [36].

.

The influence of several compounds has been investigated in both human cell lines and human subjects. In the former, HepG2 cells were treated with various concentrations of *Graptopetalum paraguayense* extracts for 48 h, with those extracts prepared in 50% ethanol in water being found to be the most effective at increasing PON1 arylesterase activity and gene expression [42]. In the latter approach, PON1 activity was considered as a marker of antioxidant protection. In this regard, administration of 0.5 L of *Ilex paraguariensis* extract (2–20 µL/mL), the polyphenol-rich beverage mate, to healthy volunteers resulted in increased PON1 activity [37]. This action could be due to 5-caffeoylquinic acid, which is the main compound in coffee, many fruits, and *Ilex paraguariensis* extracts, which showed a concentration-dependent inhibition of HDL oxidation that was complete at 25 µmol/L [43]. Begcevic et al. found that administration of cranberry extract (2 g/day) and vitamin C plus zinc (300 mg/day) to 31 healthy volunteers for four weeks induced a significant increase in PON1 activity in nonsmokers [38]. However, the administration of 300 mg/day of phenolic compounds obtained from French oak wood (*Quercus robur*) had no significant influence on glutathione levels and PON1 activities towards either arylester or lactone substrates despite a significant negative correlation between paraoxonase lactonase activity and homocysteine levels in 20 healthy volunteers [44]

Ginger (*Zingiber officinale*) has been shown to contain biologically active compounds, including gingerol, shogaol, paradol, and zingerone. In a double-blind, placebo-controlled, randomized clinical trial, supplementation of 3 g of powdered ginger for three months improved PON1 activity in patients with type 2 diabetes [39]. In this type of patients, a hydrophilic extract of *Salvia miltiorrhiza* administered for 60 days also increased PON1 activity and the latter was accompanied by a decrease in oxidative stress [40]. In mild hyperlipidemic patients, consumption of *Origanum onites* distillate significantly increased serum paraoxonase and arylesterase activities and improved endothelial function [41]. These observations point to the potential complementary role of dietary supplements in improving PON1 and their possible introduction into primary prevention of atherosclerosis. Moreover, the disparity of experimental settings indicates that more comprehensive studies are required to establish the protective effect of different plant preparations and limitations in terms of doses or pre-existing pathological damage.

## 4. Pomegranate Juice

Pomegranate juice contains tannins and anthocyanins, some of which, including punicalagin, punicalain, gallic acid, and urolithins A and B, are very powerful antioxidants [45]. For this reason, the effects of pomegranate juice have been widely studied in a range of experimental settings [46,47,48,49,50]. As such, a special section will be devoted to this juice.

In patients with carotid artery stenosis, pomegranate juice consumption for a period of 12 months resulted in an 83% increase in serum PON1 activity, together with a reduction in common carotid intima-media thickness and systolic blood pressure. No further benefit was observed when a longer administration regimen was followed [46]. Pomegranate juice consumption by diabetic patients resulted in anti-oxidative effects on serum and macrophages, with an increase in PON1 activity that reached the values found for normal subjects. These effects could contribute to an attenuation of atherosclerosis development in these patients [47]. In diabetic patients, HDL-associated PON1 arylesterase, paraoxonase, and lactonase activity increased significantly after consumption of Wonderful brand pomegranate juice (50 mL/day for four weeks). PON1 protein binding to HDL was significantly increased, and the enzyme became more stable. A similar pattern was observed in both male and female patients who consumed pomegranate polyphenol extract (5 mL/day for six weeks), although to a lesser degree in the latter [51]. A higher dose (10 mL/day) pomegranate extract for 12 weeks was found to increase PON1 in patients with active rheumatoid arthritis [48]. In hemodialyzed patients, daily administration of 1000 mg of a purified pomegranate polyphenol extract for six months increased PON1 activity [52]. Pomegranate juice, or its purified phenolic compounds punicalagin, gallic acid, or ellagic acid, promote binding of a labeled recombinant PON1 to HDL in vitro [49]. Since the association of PON1 with high-density lipoprotein (HDL) stabilizes the enzyme, these beneficial effects of pomegranate consumption on serum PON1 stability and activity could lead to retardation of atherosclerosis. Pomegranate extracts administered to Apo-E deficient mice increased serum PON1 activity, with whole fruit juice being more efficient than administration of aryls [50].

This extract has also been combined with others to improve its properties. Thus, the combination of pomegranate with date extract, which is also a good source of phenolic compounds, has been tested in *Apoe*-deficient mice. At a dose of 0.5 μmol/day/mouse of gallic acid equivalents, pomegranate juice, Hallawi date extract, date seed extract, or a combination of them were tested for three weeks. Consumption of the combination resulted in the highest increases in serum and aorta PON1 activities, the latter of which was associated with a reduction in aortic lipid peroxide content [45].

## 5. Phenolic Compounds

Cinnamate is a widespread secondary metabolite of phenolic compounds synthesized by plants. When two structurally related derivatives, namely 4-hydroxycinnamate and 3-(4-hydroxyphenyl)propionic acid, were administered at a dose of 1.35 mmol/kg to male rats fed a high-cholesterol diet, plasma PON1 activity was found to be higher in the cinnamate-derivative-supplemented groups than in the control group [53].

Curcumin is a common dietary supplement that has been shown to induce PON1 transactivation in Huh7 cells in a dose-dependent manner. However, dietary supplementation of female B6C3F1 mice with curcumin (500 mg/kg diet) for two weeks did not increase hepatic *Pon1* mRNA and protein levels. In conclusion, curcumin may be a potent PON1 inducer in cultured cells in vitro, but not in the liver of curcumin-fed mice because of its low concentrations in vivo [54]. This low bioavailability could be due to its chemical instability at the extreme pH values found in the stomach and intestine.

Resveratrol is one of the best-studied phenolic compounds found in red wine, and its influence has been tested in vitro and in vivo. Thus, incubation of HUH7 liver cells stably transfected with PON1 in the presence of 10 and 25 µmol/L resveratrol significantly increased PON1 transactivation. An ascorbic acid concentration of 1000 µmol/L slightly enhanced PON1 transactivation. Furthermore, resveratrol induces PON1 protein levels in HUH7 cells, and this induction was not counteracted by ascorbic acid [55].

To investigate the anti-atherogenic properties of resveratrol supplements (0.02% and 0.06%, *w*/*w*), *Apoe*-deficient mice were used. Plasma PON activity was significantly higher in the group receiving 0.06% resveratrol compared to the control group, as was HDL concentration and the HDL/total cholesterol ratio. Moreover, PON1 activity was negatively correlated with serum thiobarbituric acid reactive substances (TBARS) concentration [56]. Resveratrol showed a different pattern in cystathionine beta synthase-deficient mice, with a one-month administration of 0.001% resveratrol significantly decreasing serum PON1 activity [57].

Several epidemiological studies have reported that consumption of flavonoids, which are polyphenolic compounds available in plant-based foods, and especially catechins, might have a chemopreventive effect against cancer and cardiovascular disease [58]. Given these findings, their effect on PON1 has also been tested. Thus, supplementation with naringenin, or its synthetic derivative naringenin 7-*O*-cetyl ether (0.73 mmol/kg diet), increased plasma PON1 activity in high-cholesterol-fed male Sprague–Dawley rats [59]. In male Wistar rats receiving a 400 mg/kg dose of rutin for two weeks, the hepatic activity of PON1 increased by 17% compared to controls [60]. In this animal model, Jaiswal et al. showed that administration of quercetin, and to a lesser extent catechin, significantly attenuated the adverse effects of HgCl_2_ by upregulating PON1 activity and protected against LDL oxidation and lipid peroxidation [28]. A similar result was found by Mohammadshahi et al., who also observed a recovery in the decreased PON1 activity in arthritic rats when administering soy isoflavones (genistein and daidzein) for 50 days [33]. In hemodialyzed patients receiving catechins (455 mg/day), PON1 activity was higher than in patients with the placebo [61].

Genistein was the most potent flavonoid in terms of PON1-inducing activity in stably transfected Huh7 liver cells in comparison with other flavonoids. In contrast, dietary genistein supplementation (2 g/kg diet over three weeks) in growing rats did not increase hepatic *Pon1* mRNA and protein levels or plasma PON1 activity. Thus, genistein may be a PON1 inducer in cultured hepatocytes in vitro, but not in rats in vivo [62]. Plasma PON1 activity did not change after genistein was combined with polysaccharide supplementation in postmenopausal Korean women. However, the activity of other antioxidant enzymes, such as glutathione peroxidase, was significantly increased. These findings suggest that this supplementation may improve antioxidant status in postmenopausal women, but not in a PON1-dependent manner [63]. Due to the metabolism of these compounds by intestinal microbiota, a wide range of biological responses associated with different microbiomes is to be expected. Thud, daidzein may be transformed into equol, an isoflavandiol actin that acts as a non-steroidal estrogen [64]. The different effects of in vivo administration of genistein are therefore not surprising. Thus, while Mohammadshahi et al. found a marked recovery in PON1 activity [33], Schrader et al. did not observe any such changes [62]. However, there are major differences between these two papers despite using the same animal model (rat), including dose (20 in the former vs. 1 mg/kg in the latter), length of administration (50 vs. 22 days), and presence or absence of a pathological condition decreasing PON1 activity. According to our comparison of dose and observed effect, this isolated compound may have a noticeable effect on PON1 in vivo (Table 2). The change induced by tea catechins is also remarkable. Thus, flavonoids are considered to be important agents for increasing PON1 activity.

The effect of anthocyanins on HDL–PON1 activity and cholesterol efflux capacity was evaluated in hypercholesterolemic subjects, who received 160 mg of anthocyanins twice daily for 24 weeks in a double-blind, randomized, placebo-controlled trial. Anthocyanin consumption significantly increased HDL cholesterol and HDL–PON1 activity, with negative correlations being established between the latter and HDL lipid hydroperoxides. Furthermore, a strongly positive correlation was noted between HDL–PON1 activity and cholesterol efflux capacity both before and after adjustment for HDL cholesterol and APOA1 in anthocyanin-treated subjects. These results indicate that HDL–PON1 activity might participate in the antioxidant and cholesterol efflux properties of HDL [65].

## 6. Vitamins

Two vitamins, namely C and E, have received particular attention. Thus, supplementation with vitamin C provoked an increase in PON1 activity and a decrease in advanced glycation products and lipid hydroperoxide levels in hemodialysis patients. These findings provide further evidence that lipid peroxidation and impairment of the antioxidant system in the plasma of these patients may play a role in renal disease and suggest that evaluation of PON1 activity could represent a useful approach for monitoring antioxidant treatment and new dialysis therapies [66]. In a similar study, serum PON1 activity was found to decrease from preconception to labor in 35 women. A direct association between vitamin C intake and PON1 at week 32 was observed, suggesting that vitamin C supplementation in pregnant women deserves further attention [67]. However, a vitamin supplement (200–300 mg calcium, 15 mg zinc, 17 mg iron, 400 µg folic acid, 400 IU vitamin D, 70 mg vitamin C, 3 mg thiamine, 2 mg riboflavin, 20 mg niacin, 6 µg vitamin B12, and 10 mg vitamin E) had no effect on the oxidative stress status of healthy pregnant women [68]. When Begcevic et al. analyzed the effects of vitamin C and zinc (300 mg/day) on serum PON1 activity in 31 healthy volunteers over four weeks, they observed a significant increase in PON1 activity in nonsmokers after the intervention [38]. Using the commercial preparation ALA nerv^®^, which contains essential fatty acids, vitamins, and oligo-elements, Manolescu et al. reported an increase in PON1 lactonase activity in 28 post-acute stroke patients receiving two pills/day for two weeks [69].

Vitamin E supplementation increased the serum paraoxonase and arylesterase activities of propylthiouracil-induced hypothyroidism in male Sprague–Dawley rats [70]. This vitamin also recovered the decrease in PON1 induced by exercise in untrained dogs [71]. Likewise, oral supplementation of vitamin E (200 mg/day) prevented the exercise-induced decrease in PON1/Aryl esterase activity in basketball players [72]. This vitamin has been combined with other agents to improve PON1 properties. Thus, oral supplementation with vitamin E (300 mg/kg) and sodium selenite (0.5 mg/kg) once a day for four weeks in streptozotocin-induced diabetic rats restored PON1 activity to the levels observed in healthy animals [73]. The administration of 0.2% vitamin E together with 0.02% ferulic acid in *Apoe*-deficient mice also increased hepatic PON1 [74]. Other combinations, containing vitamin C, zinc gluconate, and selenium, have been prepared and tested in elite women athletes. Significant positive correlations between antioxidant defense and lipid status were found, with a significant positive correlation between PON1 activity toward paraoxon and LDL-cholesterol, but only before training and supplementation. Free-radical generation before and after training did not affect paroxonase activity, and no significant correlation was found with oxidative stress parameters [75,76].

## 7. Protein and Amino Acids

Relatively little attention has been devoted to the influence of the quality of proteins or amino acids on PON1 activity. In this regard, commercial whey protein was found to improve PON1 activity in rats compared with casein [77]. Administration of the amino acid derivative L-carnitine recovered the exercise-induced decrease in PON1 in sedentary people [78], although the non-proteinogenic amino acid taurine was found to be more active. Thus, serum paraoxonase and arylesterase activities were increased in a dose-dependent manner in taurine-treated hypothyroid Sprague–Dawley rats, and taurine concentrations were positively correlated with enzyme activities [79].

## 8. Dietary Lipids

### 8.1. Cholesterol Supplementation

Administration of cholesterol (3.6 g/kg/day) for three months decreased hepatic PON1 activity in Sprague–Dawley rats [80]. Similar results were obtained by our group using male *Apoe*-deficient mice and with lower cholesterol intakes (1 g/kg/day). We also observed a selective sex response, with females being more resistant to the action of this compound [81].

### 8.2. Polyunsaturated Fatty Acids

A combination of red yeast rice and rice bran oil increased serum PON1 levels in male Sprague–Dawley rats compared to those receiving a hypercholesterolemic diet alone [17].

The ALA nerv^®^ nutritional preparation, which contains linoleic acid, α-lipoic acid, and gamma linoleic acid along with other vitamins and selenium, provoked a significant increase in PON1 lactonase activity when evaluated in post-acute stroke patients (two pills/day for two weeks) [69].

#### Fish Oil

The influence of supplementation with this oil on PON1 has been widely studied. Thus, an increase in serum PON1 activity associated with HDL lipid composition changes was observed in obese rats receiving 20% sardine byproduct oil [82], whereas dietary administration of ω-3 fatty acids (EPA + DHA, 1.2:1) at doses of 0.3 or 1 g/kg body weight for four weeks in rats decreased the activity of PON1. A similar decrease was observed in a postprandial approach involving fish oil supplementation in Balb/C mice associated with oxidative stress. This effect was corrected in PON1-transgenic mice, thereby suggesting a PON1 lipase-like activity on chylomicron triacylglycerols [10]. However, a higher dose of ω-3 fatty acids resulted in a 45% increase in PON1 activity [83]. Hepatic PON1 activity significantly increased in fish-oil-treated rats [84].

In a double-blind randomized controlled trial carried out in 90 female patients with rheumatoid arthritis receiving 1 g of fish oil or a placebo in addition to conventional treatment, fish oil supplementation significantly increased PON1 activity [85]. Further positive evidence was obtained when comparing the HDL proteome before and after five weeks of a ω-3 PUFA-supplemented diet in smoker subjects. In that study, Burillo et al. reported that the supplement increased PON1 protein, thereby suggesting a positive change in the functionality of the HDL molecule [86]. Contrary to these findings, in a randomized controlled trial, women with iron deficiency anemia randomly assigned to receive 500 mg of DHA supplement or placebo with an iron tablet, once daily for 12 weeks, showed no significant differences in terms of serum PON1 concentration [87]. Four years ago, Whelan et al. reviewed the effect of stearidonic acid, a highly unsaturated (n-3) PUFA that is effectively metabolized to eicopentaenoic acid, and concluded that its administration is a favorable modulator of PON1 [88].

### 8.3. Olive Oil and Coenzyme Q_10_

In subjects receiving 20 mL of extra virgin olive oil supplemented with 20 or 40 mg of coenzyme Q10, a positive correlation was observed between concentration of the coenzyme in HDL and PON-1 activity, with a lower susceptibility of LDL to peroxidation also being found [89].

### 8.4. Hydrocarbons

Administration of squalene at a dose of 1 g/kg for 11 weeks showed decreased reactive oxygen species in lipoprotein fractions irrespective of the animal background (wild-type, *Apoa1*-, and *Apoe-*deficient mice) and caused a specific increase in HDL-cholesterol levels, accompanied by an increase in PON1. The increases in HDL–PON1 resulted in decreased plasma malondialdehyde levels depending on the presence of apolipoprotein A1. The phenotype related to apolipoproteins A1 and E may be particularly relevant [90].

Astaxanthin is a carotenoid pigment present in crustaceans and salmon. In soccer players, there was a significant interaction between the effect of astaxanthin supplementation and training on PON1 activity. Thus, PON1 activity increased in the supplemented group after 90 days, as did the thiol content. As such, astaxanthin was proposed to improve PON1 activity by protecting free thiol groups against oxidative modification [91].

## 9. Oligoelement Supplementation

Two oligoelements, namely selenium and zinc, have received particular attention. With regard to the former, four months of selenium administration at 1 mg/kg diet improved the high-fat-diet-mediated reduction of serum PON1 enzyme activity and PON1 protein levels, with no changes in hepatic mRNA expression, in male Sprague–Dawley rats [92]. However, dietary supplementation with 1 or 3 mg Se/kg diet, with or without 250 or 750 mg vitamin E/kg diet, did not completely abolish the effects of MeHg as regards a reduction in PON1 activity [93]. Inclusion of ALA nerv^®^, a nutritional supplement containing selenium (two pills/day for two weeks), resulted in a significant increase in PON lactonase activity when evaluated in post-acute stroke patients [69].

Zinc administration partially reversed the significant decrease in PON1 induced in rats by administration of carbon tetrachloride [94] and in mice by *Schistosoma mansoni* infection [95]. When tested in hemodialysis patients, a 100 mg/day zinc supplementation for two months significantly increased serum levels of PON activity [96]. In 31 healthy volunteers exposed to commercially available preparations of vitamin C and zinc (300 mg/day) for four weeks, Begcevic et al. also reported a significant increase in PON1 activity in nonsmokers after the intervention [38]. In contrast, mineral (15 mg zinc, 17 mg iron) and vitamin supplementation failed to increase PON1 activity in pregnant women [68]. It has also been proposed that copper supplements may exert a protective effect on PON1, assuming that a copper deficiency decreases this enzyme [97]. The results suggest that the doses and/or exclusion criteria for subjects (smokers or non-smokers) and types of patients may be crucial in the outcome and should be carefully tested.

## 10. Others

Beta glucan supplementation increased PON1 activities in the brain and sciatic nerve of streptozotocin-induced diabetic rats [98]. Dietary supplements of symbiotics (Perfectin) and probiotics (Protexin) induced increased PON1 activity in White Leghorn chicken [99]. Supplementation with the commercial formulas Build-up (Nestle) and Maxijul (SHS Ltd.) for eight weeks failed to recover the decreased PON activity observed in frail elderly patients [100].

## 11. Conditions Negatively Affecting PON1 Activity

Several dietary supplements have been identified to have a negative influence on PON1 activity, including quercetin, vitamin, and amino acid supplementations, or at least in certain genetic backgrounds, animal models, or pathological conditions. As mentioned in Section 2, the response of PON1 activity to dietary supplements is modified by *APOE* gene polymorphisms. In this regard, the administration of a diet containing quercetin resulted in lower hepatic mRNA and protein levels of PON1 in *APOE4* than in *APOE3* transgenic mice [19]. Moreover, vitamin E supplementation in high-fat and -cholesterol diets significantly reduced serum PON1 activity in baboons [101]. Supplementation of the amino acid derivative betaine had no effect on plasma PON1 paraoxonase or arylesterase activities in hyperhomocysteinemic mice. However, betaine- and methionine-restricted diets decreased paraoxonase activity in this type of human patient [102].

## 12. PON2

Paraoxonase 2 (PON2), a member of the paraoxonase gene family, has been shown to protect macrophages against oxidative stress. Thus, incubation of J774A.1 macrophages with pomegranate juice (0–50 µM of total polyphenols) increased expression (mRNA, protein) and PON2 activity, and reduced macrophage oxidative status, in a dose-dependent manner. These effects could be attributed to punicalagin and gallic acid, which are the main phenolic components of this juice. The polyphenol-induced upregulation of PON2 was inhibited by using the peroxisome proliferator-activated receptor gamma (PPARγ) inhibitor GW9662. Similarly, the PPARγ ligand rosiglitazone stimulated macrophage PON2 expression in a dose-dependent manner. Inhibition of AP-1 activation with SP600125 attenuated pomegranate juice-induced upregulation of PON2. Thus, the pomegranate juice stimulatory effect on macrophage PON2 expression was associated with activation of the transcription factors PPARγ and AP-1 [103]. Consumption of whole fruit juice and peels, either as a liquid or powder extract, resulted in a significant increase in PON2 lactonase activity in mice macrophages [50]. Another phenolic-rich plant has been tested as regards its ability to modulate PON2. Thus, in vitro incubation of THP-1 macrophages with yerba mate (*Ilex paraguariensis*) extract increased *PON2* gene expression at concentrations ranging from 1 to 3 µmol/L, whereas higher concentrations only increased the activity. Increased *PON2* gene expression and activity were observed in the monocytes of healthy women 2 h after yerba mate consumption. However, when this extract was administered for seven days, the increase in gene expression was not reflected in the activity of either monocytes or monocyte-derived macrophages [104].

## 13. PON3

Despite having an important protective role against atherosclerosis, no studies have been found regarding dietary regulation of the third member of this family [6].

## 14. Conclusions and Outlook

A wide range of experimental evidence indicates that increasing PON1 and PON2 activity may decrease oxidative stress and contribute to delaying the outcome of chronic diseases or mitigating their impact on quality-of-life in patients. The search for plant extracts that are able to modulate these activities is a highly active field of research, as reflected in this review. Given the tremendous amount of work required to characterize planetary diversity in this regard, this field will undoubtedly last for many more years. Complete chemical characterization of these plant extracts is an urgent and demanding task as regards both identifying the target molecule and determining the matrix that protects the active component. High-throughput screening will need to be carried out in cell culture for obvious reasons of efficiency and animal welfare. To date, pomegranate juice has been well characterized, and *Aronia melanocarpa* extract seems very promising. The translation of these more promising findings into in vivo settings needs to be done carefully, paying special attention to formulation and stability, doses, length of administration, and selection of the experimental model. Once these aspects have been resolved, a wide range of formulations can be envisioned for nutraceuticals and functional foods to enhance the protective role of paraoxonases.

## Figures and Tables

**Table 1:** Plant preparations found to increase serum PON1 in different experimental designs.

Extract	Experimental Model	Dose	Effect	References
*Eucommia ulmoides* Oliver leaf	Diabetic C57BL/KsJ-db/db mice	400 mg/kg bw	↑ 22%	[21]
*Murraya koenigii*	Streptozotocin-induced diabetic mice	150 mg/kg	↑ 105%	[22]
Grape seed extracts	Streptozotocin-induced diabetic rats	100 mg/kg	↑ 86%	[23]
Red wine polyphenol extract	Heterozygous *Cbs*-deficient mice	100 mg/kg	↑ 20%	[25]
*Sambucus nigra*	*Apoe*-deficient mice	200 mg/kg	↑ 20%	[26]
*Aronia melanocarpa*	*Apoe*-deficient mice	6 mg/kg	↑ 39%	[27]
Onion extract	Mercuric chloride-induced oxidative insult in male Wistar rats	20 mg/kg	↑ 30%	[28]
*Aronia melanocarpa*	Rats on a high-fructose and high-fat diet	Not reported	↑ 65%	[29]
Cornelian cherry	Rats on a high-fructose and high-fat diets	Not reported	↑ 45%	[30]
Genistein	Arthritic rats	20 mg/kg	↑ 230%	[33]
*Euterpe oleracea* Mart	Female Fischer rats on high-cholesterol, high-fat diets	2 mg/kg	↑ 60%	[34]
Avocado	Male Wistar rats	28 g/kg	↑ 33%	[35]
*Ilex paraguariensis*	Healthy volunteers	0.5 L of extract	↑ 10%	[37]
Cranberry extract with vitamin C and zinc	Healthy volunteers	2 g/day (300 mg/day)	↑ 67%	[38]
*Zingiber officinale*	Type 2 diabetic patients	3 g	↑ 28%	[39]
*Salvia miltiorrhiza*	Type 2 diabetic patients	Not reported	Increased PON1 activity	[40]
*Origanum onites*	Hyperlipidemic patients	Not reported	↑ 14%	[41]

bw: body weight.

**Table 2:** Effect of phenolic compounds on PON1 in different in vivo experimental settings.

Compound	Experimental Model	Dose	Effect	References
3-(4-Hydroxyphenyl) propionic acid	Rats fed a high-cholesterol diet	1.35 mmol/kg	↑	[53]
Resveratrol	*Apoe*-deficient mice	12 mg/kg	↑75%	[56]
Flavonoids				
Naringenin	High-cholesterol-fed Sprague–Dawley rats	4 mg/kg	↑ 37%	[59]
Rutin	Wistar rats	400 mg/kg	↑ 17%	[60]
Quercetin	HgCl_2_ treated Wistar rats	20 mg/kg	↑ 20%	[28]
Genistein	Arthritic rats	20 mg/kg	↑ 230%	[33]
Tea catechins	Hemodialyzed patients	6 mg/kg	↑ 150%	[61]
Anthocyanins	Hypercholesterolemic subjects	4 mg/kg	↑ 22%	[65]
